# Community-Acquired Acute Kidney Injury and Late Kidney Dysfunction in Survivors of COVID-19 Hospitalization

**DOI:** 10.1016/j.ekir.2025.06.048

**Published:** 2025-07-03

**Authors:** Heitor S. Ribeiro, Marcella M. Frediani, Lia Marçal, Leila Antonângelo, Guilherme S. Catharina, Luis Yu, Dirce M.T. Zanetta, Thais Mauad, Tiana C.L. Moreira, Nelson Gouveia, Geraldo F. Busatto, Carlos R.R. Carvalho, Emmanuel A. Burdmann, HCFMUSP COVID-19 Study Group

**Affiliations:** 1 Departamento de Clínica Médica, LIM 12, Laboratório de Pesquisa Básica em Doenças Renais, Hospital das Clinicas HCFMUSP, Universidade de Sao Paulo, Sao Paulo, Brazil; 2 Departamento de Patologia, LIM 03, Laboratório de Medicina Laboratorial, Faculdade de Medicina, Hospital das Clinicas HCFMUSP, Universidade de São Paulo, Sao Paulo, Brazil; 3 Departamento de Epidemiologia, Faculdade de Saude Publica, Universidade de Sao Paulo, Sao Paulo, Brazil; 4 Departamento de Patologia, LIM 05, Laboratório de Poluição Atmosférica Experimental, Hospital das Clinicas HCFMUSP, Faculdade de Medicina, Universidade de Sao Paulo, Sao Paulo, Brazil; 5 Departamento de Medicina Preventiva, Hospital das Clinicas HCFMUSP, Faculdade de Medicina, Universidade de Sao Paulo, Sao Paulo, Brazil; 6 Departamento e Instituto de Psiquiatria, Hospital das Clinicas HCFMUSP, Faculdade de Medicina, Universidade de São Paulo, Sao Paulo, Brazil; 7 Divisão de Pneumologia, Instituto do Coração, Hospital das Clinicas HCFMUSP, Faculdade de Medicina, Universidade de Sao Paulo, Sao Paulo, Brazil

**Keywords:** acute kidney injury, chronic kidney disease, dialysis, kidney replacement therapy, post-acute COVID-19 syndrome, SARS-CoV-2

## Abstract

**Introduction:**

Data on the incidence and risk factors for renal long COVID are scarce. We aimed to investigate 2 acute kidney injury (AKI) phenotypes, namely community-acquired (CA; CA-AKI) and hospital-acquired (HA; HA-AKI), and the development of late kidney dysfunction in survivors of COVID-19 hospitalization.

**Methods:**

This is a prospective cohort study of survivors of moderate-to-severe COVID-19 hospitalization in Brazil, from March to August 2020. The patients were assessed for up to 11 months after hospital discharge. Exposure was CA-AKI and HA-AKI. The main outcome was kidney dysfunction defined as incident low estimated glomerular filtration rate (eGFR; < 60 ml/min per 1.73 m^2^) and/or eGFR decline ≥ 25% from discharge at follow-up. An adjusted binary logistic regression analysis was run.

**Results:**

A total of 655 survivors were evaluated (6.5 ± 1.9 follow-up months); 79% had AKI (35% CA and 43% HA); 14% used kidney replacement therapy (KRT). Late kidney dysfunction occurred in 28% of the patients (16% with incident low eGFR and 27% with eGFR decline ≥ 25%). CA-AKI, but not HA-AKI, was independently associated with late kidney dysfunction (adjusted odds ratio [aOR] = 7.3, 95% confidence interval (CI): 3.6–15.8 and aOR = 2.2, 95% CI: 0.9–4.8, respectively).

**Conclusion:**

In conclusion, late kidney dysfunction affected 1 in 4 COVID-19 survivors. CA-AKI, but not HA-AKI, was an independent risk factor for late kidney dysfunction. These findings suggest that renal long COVID might be frequent and that a specific AKI phenotype (CA-AKI) may play a crucial role in its development. Our research highlights the need for CA-AKI prevention and for the long-term follow-up and care of patients affected by this AKI phenotype during COVID-19 infection.

## Graphical abstract

**Figure:**
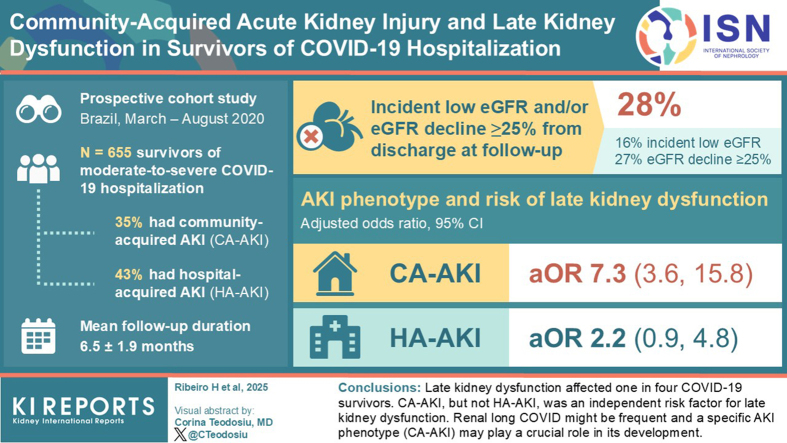

The infectious COVID-19 is associated with short- and long-term adverse outcomes.^1^^,^^2^ AKI is a major short-term outcome, affecting 29% of patients, with 47% of these cases resulting in death.^3^ The long-term outcomes, referred to as postacute sequelae of SARS-CoV-2 infection or long COVID have become a priority in the management and care of COVID-19 survivors.^4^^,^^5^ Long-term follow-up and screening for multiple organs, including the kidneys, have been advocated for COVID-19 survivors because of the likelihood of a public health crisis in the future.^6^^,^^7^

A large constellation of organ dysfunction and injury has been described in patients with long COVID, including kidney dysfunction.^8^, ^9^, ^10^ Previous findings from our cohort showed that clinical, sociodemographic, and environmental factors significantly impact post–COVID-19 syndrome^11^; however, kidney-related outcomes have yet to be explored.

Although kidney involvement during the acute phase of COVID-19 has been frequently described,^12^, ^13^, ^14^ renal long COVID, differently from other organs, has been less investigated. Previous studies showed very heterogeneous findings,^15^, ^16^, ^17^, ^18^ with chronic kidney disease (CKD) development ranging from 0.4%^19^ to 49%,^20^ depending on the studied population. Among the potential risk factors for late kidney dysfunction in long COVID, available evidence suggests that AKI may be independently associated with its development.^21^ Outside the COVID-19 context, previous evidence showed that different AKI phenotypes, such as CA versus HA, present distinct short- and long-term outcomes.^22^^,^^23^ However, no study to date has investigated these phenotypes in the context of long COVID. Moreover, the majority of the studies on renal long COVID are retrospective and assessed only the eGFR,^15^, ^16^, ^17^^,^^24^ whereas other kidney injury biomarkers remained underexplored.

To address this knowledge gap, we investigated the role of 2 AKI phenotypes, CA-AKI and HA-AKI, in the incidence of late kidney dysfunction in a prospective cohort of survivors of moderate-to-severe COVID-19 hospitalization in Brazil.

## Methods

### Study Design, Setting, and Participants

This report is part of the Hospital das Clínicas, Faculdade de Medicina, Universidade de São Paulo (HCFMUSP) COVID-19 study. The study protocol is provided in detail elsewhere.^25^ Briefly, survivors of moderate-to-severe COVID-19 hospitalization were invited for follow-up evaluation comprising teleconference interviews and in-person visits. The inclusion criteria included laboratory-confirmed COVID-19 diagnosis, hospital stay ≥ 24 hours, and age ≥ 18 years. The exclusion criteria were diagnosis of CKD under KRT, only 1 result for serum creatinine (sCr) during hospitalization, sCr not assessed at discharge or follow-up, and only teleconsulting at follow-up. All eligible survivors were included unless they declined to participate.

Research protocols were approved by the local ethics committees (#4.270.242, #4.502.334, #4.524.031, #4.302.745, and #4.391.560). All patients provided written informed consent before participation. This manuscript was written in accordance with the Strengthening the Reporting of Observational Studies in Epidemiology Statement.^26^

### Data Collection

Patients were evaluated after hospital discharge, with follow-up ranging from 3 to 11 months. This evaluation comprised a semistructured teleconsultation interview covering sociodemographic characteristics, occupational data, lifestyle habits, as well as medical history and an in-person visit for a multidisciplinary evaluation (physical assessments and laboratory tests). Data were captured and stored in real-time on the Research Electronic Data Capture (REDCap) system.

#### Clinical and Laboratory Assessments During Hospitalization and Follow-Up

Details of clinical and laboratory assessments during hospitalization and follow-up visits are provided elsewhere.^25^ Key variables relevant to this study are briefly described as follows:

• Hospital admission: COVID-19 symptom duration before hospital admission, comorbidities (kidney disease other than CKD); body mass index (obesity ≥ 30 kg/m^2^), Charlson comorbidity index, Simplified Acute Physiology Score III score; oxygen saturation; and blood laboratory (sCr, C-reactive protein, hemogram, and D-dimer) analyzed using standard methodology.
• During hospitalization: daily sCr, length of hospital stay, intensive care unit (ICU) admission and ICU length of stay, orotracheal intubation, corticoid use, and KRT use.
• Follow-up visit: blood samples were collected using ethylenediamine tetraacetic acid tubes, centrifuged, and stored at −80 °C freezers for further analysis of sCr by a specialized laboratory at the university (conducted by LA and LOM); urine samples were collected and used for assessment of urinary protein, leukocytes, and red blood cells. A urine aliquot was centrifuged and stored in a −80 °C freezer for the analysis of creatinine, albumin, and urinary kidney biomarkers. The detailed methods for the kidney urinary biomarkers analysis are described in Supplementary Methods. Events after hospital discharge (vaccination, medical appointments, and hospital readmission) were recorded.

Sociodemographic data were collected from the patients’ electronic health records or self-reported by the patient or relative. Low socioeconomic status was defined as categories CD and DE of the Issiaka Brasileira de Impresas de Pesquisa.^27^ Greenspace in the patient’s neighborhood was calculated as previously described.^11^ Further details are described in Supplementary Methods.S1–S3

### Exposure

#### AKI

Data on sCr were collected upon hospital admission, daily for up to 28 days during hospitalization, and at hospital discharge. HA-AKI was diagnosed according to the Kidney Disease: Improving Global Outcomes (KDIGO) AKI Guideline using the sCr criteria and stratified according to the KDIGO stages.^28^ A diagnosis of CA-AKI was made using a previously used modified KDIGO criteria, considering a decrease in sCr ≥ 0.3 mg/dl or ≥ 50% relative to the hospital admission value.^29^^,^^30^ KDIGO staging was not applicable for CA-AKI.

### Outcomes

#### Late Kidney Dysfunction

Late kidney dysfunction at the follow-up visit (ranging from 3 to 11 months) was defined as a composite outcome, including incident low eGFR and/or eGFR decline.

#### Incident Low eGFR

eGFR was calculated using the CKD Epidemiology Collaboration race-free sCr-based equation.^31^ An incident low eGFR was defined as < 60 ml/min per 1.73 m^2^ at follow-up in patients who did not have a low eGFR at hospital discharge.^32^

#### eGFR Decline

A decline in eGFR was defined as a reduction ≥ 25% in the follow-up visit compared with that at hospital discharge.^33^

Additional markers of abnormal kidney function at the follow-up visit included albuminuria (urinary albumin-to-creatinine ratio ≥ 30 mg/g^32^) and abnormal urine sediment (hematuria: > 3 red blood cells/field and/or leukocyturia: > 10 white blood cells/field).^32^ These additional markers were not considered for the main outcome of kidney dysfunction because of missing data and lack of assessment at hospital discharge.

### Statistical Analyses

The sample size calculation was not performed because we recruited as many survivors as possible.

#### Missing Data and Imputation

Missing data are detailed in Supplementary Table S1. No imputation was performed for kidney outcomes because they were dependent variables. For covariate variables included in the adjusted logistic regression models, we imputed missing data by applying the multiple imputation regression method, assuming data were missing completely at random.^22^ Five imputed datasets have been generated for continuous variables with < 15% missing. Age, sex (as a biological variable, not gender), and AKI phenotypes were used as predictors, whereas the variables of imputation interest were not. Frequency variables were described as valid percentages.

#### Descriptive Analysis

Continuous data are presented as mean and SD or median and interquartile range, depending on data normality, which was assessed through histogram visual inspection and the Kolmogorov-Smirnov test. Comparisons among groups with AKI (CA-KI and HA-AKI) and without AKI were conducted using the Kruskal-Wallis or the 1-way analysis of variance with Bonferroni *post hoc* correction. The unpaired *t* test or the Mann-Whitney U test were performed for comparisons between the groups with and without kidney dysfunction, depending on data normality. For categorical variables, we performed the chi-square or Fisher Exact tests.

The annual eGFR change was calculated using the formula: eGFR change (/yr) = (eGFR at follow-up − eGFR at hospital discharge) / (follow-up months / 12). This approach accounted for differences in follow-up duration across survivors. Crude eGFR change was also reported (eGFR at follow-up − eGFR at hospital discharge).

#### Association Measure

The association between AKI phenotypes (independent) and late kidney dysfunction (dependent) was tested by running binary logistic regressions in unadjusted and adjusted models. Odds ratios and 95% CI were calculated. Adjusted models included clinically relevant confounders, and variables with a *P-*value < 0.15 in the group comparison (with vs. without AKI). Two models were constructed; model 1 considered variables from the admission and included older age (reference ≤ 60 years), sex (reference = female), ethnicity (reference = White), socioeconomic status (reference = high + medium), Charlson comorbidity index (continuous), greenspace (continuous), and sCr at hospital admission (continuous). Model 2 was built in addition to model 1 and included event variables from the hospitalization period: ICU admission (reference = no admission), hospital length of stay (continuous), use of KRT (reference = no use), and eGFR at discharge (continuous). Subgroup analysis by sex was conducted.

Additional analysis was conducted to test the association of AKI severity (which could include both AKI phenotypes) with late kidney dysfunction. We stratified the cohort into no AKI (reference), AKI without use of KRT, and AKI with use of KRT. In this analysis, CA-AKI was included as a covariate in model 1 (reference = no AKI + HA-AKI). Analyses were performed using the Statistical Package for the Social Sciences (version 29.0, SPSS Inc, Chicago, IL) and GraphPad Prism (version 8.4, GraphPad Software, San Diego, CA). A 2-tailed *P-*value < 0.05 was considered statistically significant.

## Results

### Participants’ Recruitment and Characteristics

**Figure 1:**
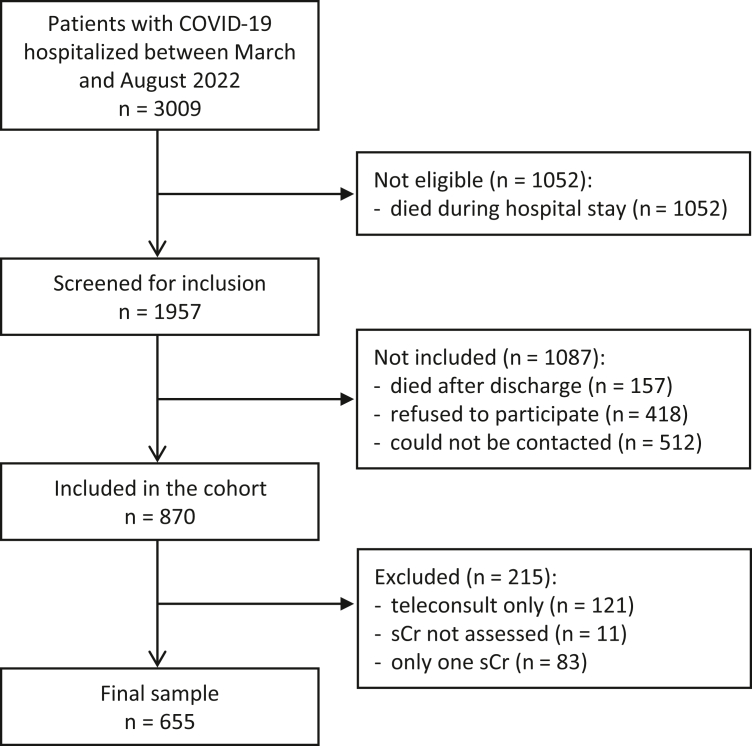
Flowchart of recruitment. sCr, serum creatinine.

A total of 1957 survivors were screened, and 870 were included in the prospective cohort. After applying the exclusion criteria, 655 participants were included in this study (Figure 1). The study participants had significantly higher body mass index, higher male prevalence, were more frequently admitted to ICU, used more orotracheal intubation, and had longer hospital stay than those who did not participate (Supplementary Table S2).

### Cohort Characterization

During follow-up after hospital discharge, 157 patients died (see Supplementary Table S3 for comparison with survivors). Among the included survivors in the study analysis (*n* = 655), most (69.5%) were assessed ≥ 6 months after hospital discharge, with a median follow-up time of 6 months (interquartile range: 5 – 8). The mean age of the cohort was 56 ± 13 years, and 45% were aged ≥ 60 years old. Female sex comprised 46% of the cohort; 46% were White, and 41% were obese. Low socioeconomic status was observed in 40%. Kidney disease other than CKD was reported in 7.5%. A total of 67% were admitted to the ICU with a median length of stay of 10 (6 – 18) days. At hospital discharge, 22% of the cohort had an eGFR < 60 ml/min per 1.73 m^2^.

### AKI Phenotypes

A total of 79% survivors had AKI during hospitalization (35% with CA-AKI and 43% with HA-AKI). Characteristics according to the AKI phenotypes are presented in Table 1. The CA-AKI group was older, had a higher Charlson comorbidity index and Simplified Acute Physiology Score III score at hospital admission, and used KRT more frequently (mainly within 48 hours from admission) compared with the HA-AKI group. Conversely, the HA-AKI group had more ICU admissions, orotracheal intubation, and longer hospital and ICU stays. Overall, only 3 patients (0.5%) were not treated with anticoagulants. At hospital discharge, the HA-AKI group had lower eGFR and a higher frequency of eGFR < 60 ml/min per 1.73 m^2^ (43.7%, *P* < 0.001) compared with the CA-AKI group.

**Table 1:** Characteristics of the cohort according to the acute kidney injury phenotypes

Variables	No AKI (*n* = 141)	Community-acquired AKI (*n* = 230)	Hospital-acquired AKI (*n* = 284)	*P*-value
Age (yrs)	52.9 ± 13.9	58.5 ± 13.0^a^^,^^b^	55.0 ± 13.9	< 0.001
Older age (≥ 60 yrs), *n* (%)	50 (35.5)	127 (55.2)	115 (40.5)	< 0.001
Female, *n* (%)	68 (48.2)	90 (39.1)	144 (50.7)	0.028
Ethnicity, *n* (%)				0.268
White	67 (47.9)	96 (42.5)	137 (49.5)	
Black	15 (10.7)	40 (17.7)	35 (12.6)	
Mixed	56 (40.0)	86 (38.1)	97 (35.0)	
Others^c^/Not reported	2 (1.4)	4 (1.7)	8 (2.9)	
Low socioeconomic status, *n* (%)	54 (38.8)	90 (40.9)	114 (40.7)	0.917
Greenspace (%), median (IQR)	37.2 (27.8–53.0]	38.1 (26.0–59.4]	30.4 (20.7–52.2]	0.053
Hospital admission, median (IQR)				
Duration of symptoms until admission (d)	8 (6–11]	8 (6–11]	8 (5–10]	0.535
sCr (mg/dl)	0.8 (0.6–0.9]	1.5 (1.1–2.2)^a^^,^^b^	0.8 (0.7–1.2]^a^	< 0.001
Charlson comorbidity index	2 (1–4)	3 (2–5)^a^^,^^b^	3 (2–4)	< 0.001
SAPS 3 score	55 (46–65]	62 (51–71)^a^^,^^b^	56 (45–68)	0.003
SpO2 < 90%, *n* (%)	21 (23.9)	49 (28.3)	62 (30.2)	0.539
Obesity, *n* (%)	37 (33.3)	71 (38.4)	111 (45.5)	0.073
Events during hospitalization, median (IQR)				
Hospital length of stay (d)	10 [7–15)	13 [8–22)^a^^,^^b^	21 (12–35)^a^	< 0.001
ICU admission, *n* (%)	71 (50.7)	137 (62.3)	221 (78.6)	< 0.001
ICU length of stay (d)	5 (3–9]	9 (6–14)^a^^,^^b^	14 (8–24)^a^	< 0.001
Intubation, *n* (%)	32 (23.0)	86 (39.4)	178 (63.3)	< 0.001
Corticoid use, *n* (%)	90 (63.8)	152 (66.1)	187 (65.8)	0.894
peak sCr (mg/dl)	0.9 (0.8–1.0)	1.6 (1.1–2.6)^a^^,^^b^	2.6 (1.5–5.9)^a^	< 0.001
KRT use, *n* (%)	0 (0)	51 (22.2)	40 (14.1)	< 0.001
Days on KRT, median [IQR]	-	14 (7–22)	10 (5 – 17)	0.169
Days from admission to KRT, *n* (%)	-	2 (1–7)	4 (1–8)	0.167
KRT use within 48 h from admission, *n* (%)	-	26 (51.0)	12 (30.0)	0.044
Kidney function at discharge				
eGFR (ml/min per 1.73 m^2^), median [IQR]	100.9 (86.3–110.6)	100.8 (84.4–111.1)^b^	66.0 (40.9–95.4)^a^	< 0.001
eGFR < 60, *n* (%)	5 (3.5)	18 (7.8)	124 (43.7)	< 0.001
Events between discharge and follow-up, *n* (%)				
COVID vaccination	12/76 (15.8)	22/111 (19.8)	20/163 (12.3)	0.235
Medical appointment	41 (29.1)	67 (29.1)	67 (23.6)	0.258
Hospital readmission	12 (8.5)	26 (11.3)	26 (9.2)	

AKI, acute kidney injury; eGFR, estimated glomerular filtration rate; ICU, intensive care unit; IQR, interquartile range; KRT, kidney replacement therapy; SAPS 3, Simplified Acute Physiology Score III; sCr, serum creatinine; SpO2, peripheral capillary oxygen saturation.

Low socioeconomic status was classified according to the Associação Brasileira de Empresas de Pesquisa (ABEP) scores C2 + DE.

a and.

b mean significant differences to the no-AKI and hospital-acquired AKI groups, respectively.

c includes indigenous and East Asian.

### Late Kidney Dysfunction

**Figure 2:**
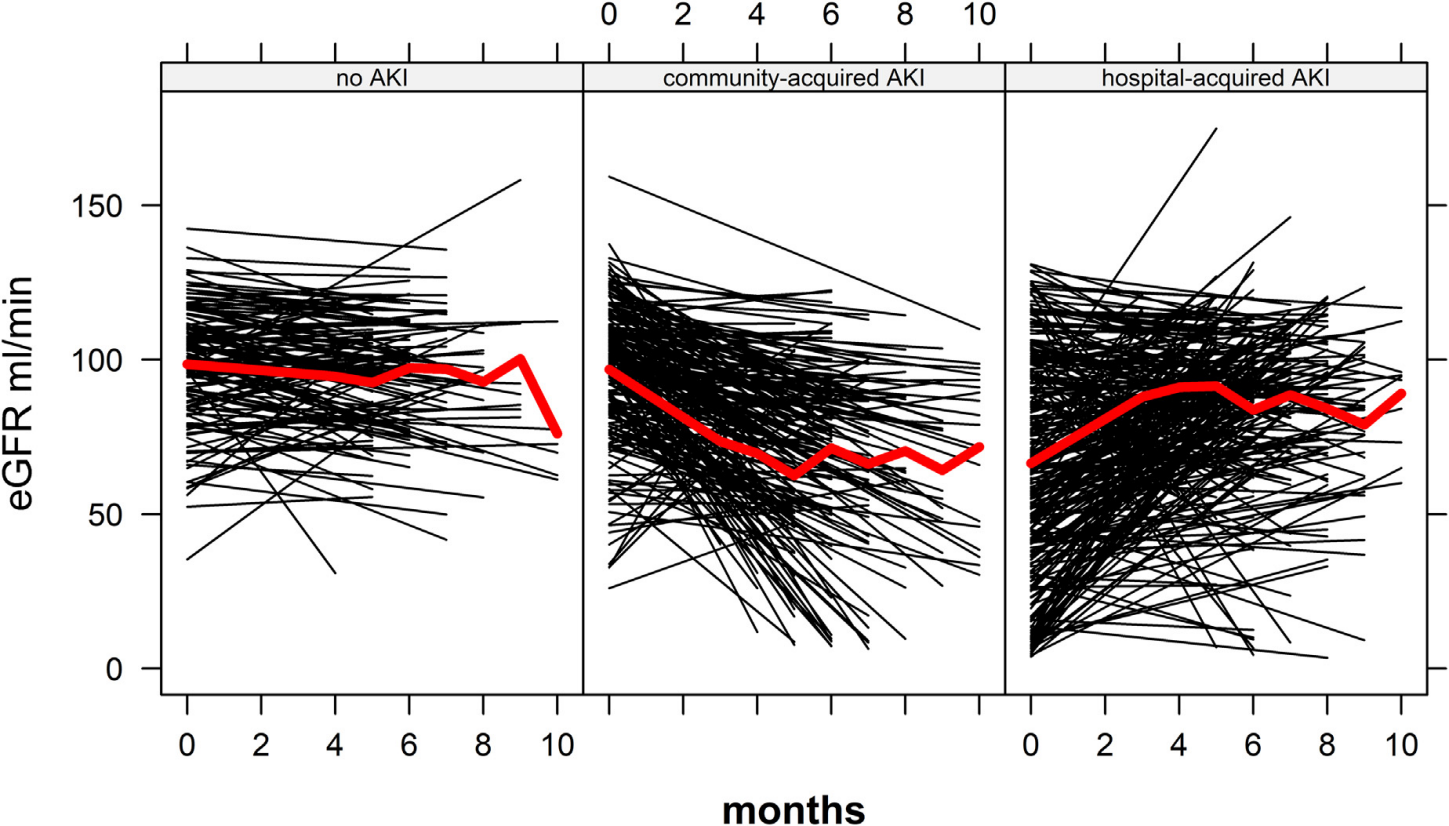
Estimated glomerular filtration rate values over time. AKI, acute kidney injury; eGFR, estimated glomerular filtration rate.

Absolute trajectories in eGFR (ml/min per 1.73 m^2^) from hospital discharge to the follow-up visit are shown in Figure 2. Annual eGFR change in the complete cohort was −4.8 ml/min per 1.73 m^2^ (95% CI: −12.0 to 2.4). In the no-AKI group, annual eGFR change was −6.9 ml/min per 1.73 m^2^ (95% CI: −13.2 to −0.6) versus −61.9 (95% CI: −73.6 to −50.3) in the CA-AKI group and 42.5 (95% CI: 32.0–53.1) in the HA-AKI group. Crude eGFR change values may be seen in Supplementary Table S4.

**Figure 3:**
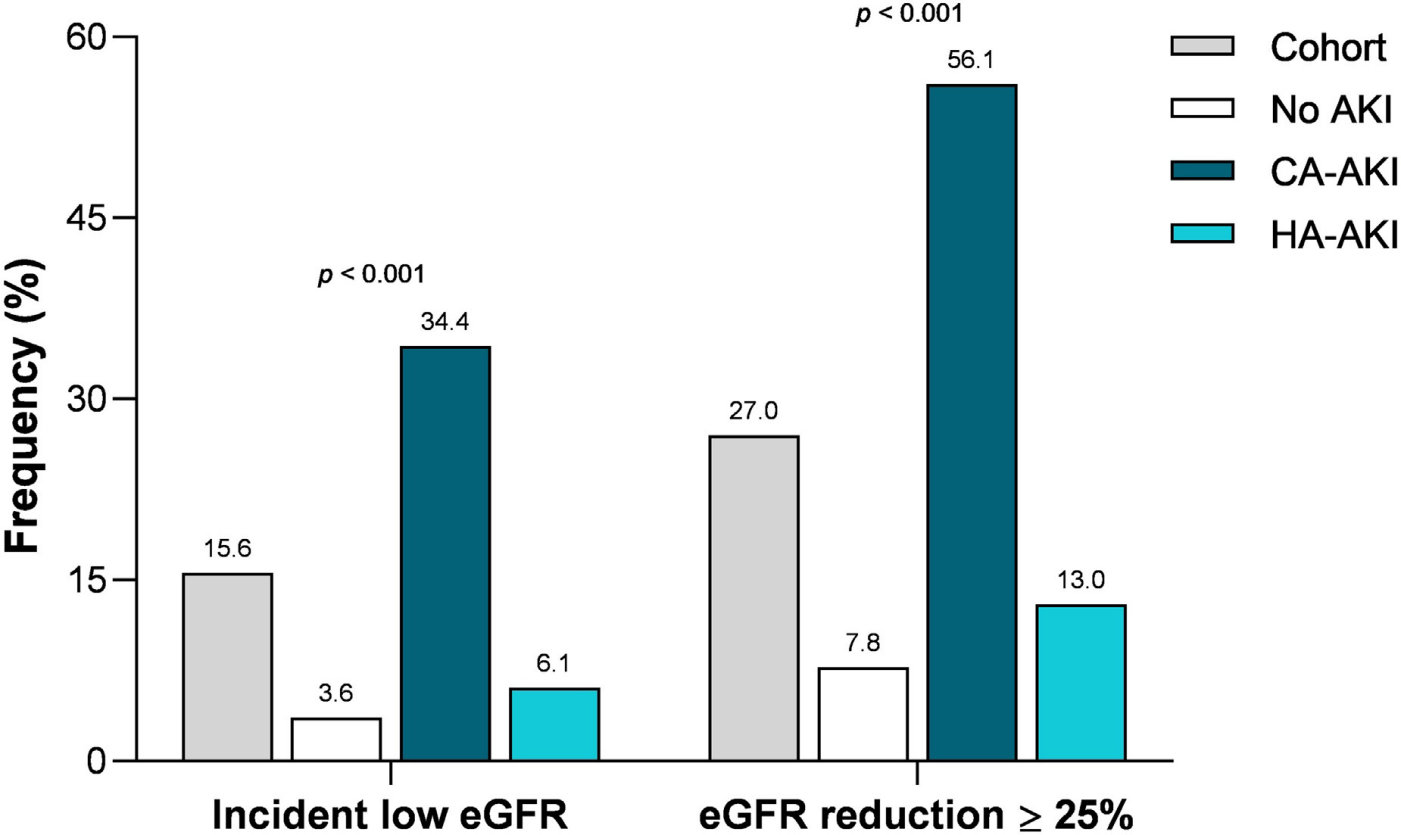
Kidney function at post–hospital discharge follow-up visit. AKI, acute kidney injury; CA-AKI, community-acquired AKI; eGFR, estimated glomerular filtration rate; HA-AKI, hospital-acquired AKI.

Overall, late kidney dysfunction was observed in 28.1% (*n* = 184) of the survivors. An incident low eGFR was present in 15.6% of patients (*n* = 97) (the total of patients with low eGFR at follow-up was 130, but 33 were discharged from hospital with an already low eGFR and therefore not considered for this analysis), whereas 27.0% (*n* = 177) showed an eGFR decline ≥ 25% (Figure 3). Overlap between outcomes was observed in 90 of 184 survivors (48.9%).

At follow-up, 29.6% of survivors (*n* = 154) exhibited albuminuria (135 missing data), and 20.4% (*n* = 130) had abnormal urine sediment (19 missing data). Among the individual components of abnormal urine sediment, hematuria was found in 9.1% (*n* = 58) and leukocyturia in 14.5% (*n* = 92). No significant difference was observed between the patients with and without kidney dysfunction for albuminuria or abnormal urine sediment (*P* = 0.359 and 0.792, respectively). The comparisons for sociodemographic and clinical variables between patients with and without late kidney dysfunction can be seen in Supplementary Table S5.

In Table 2, we show a higher proportion of patients with clusterin and trefoil factor 3 levels > the 75^th^ percentile in the late kidney dysfunction group compared with the no late kidney dysfunction (31.3% vs. 22.8%, *P* = 0.046 and 31.9% vs. 22.5%, *P* = 0.025, respectively). Continuous values can be found in Supplementary Table S6.

**Table 2:** Comparison of the urinary biomarkers above the normal value or the 75^th^ percentile in patients with and without late kidney dysfunction

Urinary biomarkers	*n*	No kidney dysfunction	Kidney dysfunction	*P*-value
NGAL ≥ 150 (ng/ml)^a^	538	34 (8.6)	20 (14.0)	0.067
KIM-1 ≥ 0.69 (ng/ml)	537	93 (23.7)	40 (27.8)	0.328
TIMP-2 ≥ 34.3 (ng/ml)	538	94 (23.9)	40 (27.8)	0.352
IGFBP-7 ≥ 1046 (ng/ml)	540	96 (24.2)	39 (27.1)	0.500
TIMP-2∗IGFBP-7/1000 ≥ 2.0 (ng/ml)^a^	538	265 (67.4)	99 (68.8)	0.772
Clusterin ≥ 49.7 (ng/ml)	538	90 (22.8)	45 (31.3)	0.046
MCP-1 ≥ 0.44 (ng/ml)	540	90 (22.7)	42 (29.2)	0.124
Interleukin-18 ≥ 141.1 (pg/ml)	503	90 (24.5)	28 (20.6)	0.355
Glutathione S-transferase ≥ 26.1 (pg/ml)	533	95 (24.3)	38 (26.8)	0.561
β-2 microglobulin ≥ 101.1 (ng/ml)	526	94 (24.0)	37 (27.4)	0.436
Cystatin C ≥ 31.7 (ng/ml)	524	102 (26.2)	28 (20.7)	0.204
Trefoil factor 3 ≥ 1364 (ng/ml)	540	89 (22.5)	46 (31.9)	0.025

IGFBP-7, insulin-like growth factor-binding protein-7; KIM-1, kidney injury molecule 1; MCP-1, monocyte chemoattractant protein-1; NGAL, neutrophil gelatinase-associated lipocalin; TIMP-2, tissue inhibitor metalloproteinase 2.

Values are reported as absolute and relative (%) based on the 75^th^ percentile, unless otherwise stated.

a Cut-off value recommended by the manufacturer.

### Association Between the 2 AKI Phenotypes and Late Kidney Dysfunction

**Figure 4:**
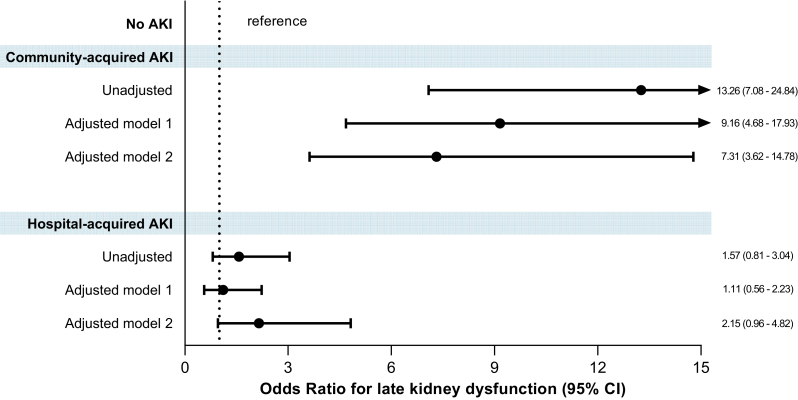
Association between the two studied acute kidney injury phenotypes and late kidney dysfunction. Adjusted model 1 included older age (reference = < 60 years), sex (reference = female), ethnicity (reference = White), socioeconomic status (reference = high + medium), Charlson comorbidity index (continuous), greenspace (continuous), and sCr at hospital admission (continuous). Adjusted model 2 included model 1 in addition to ICU admission (reference = no admission), hospital length of stay (continuous), use of kidney replacement therapy (reference = no use), and eGFR at discharge (continuous). AKI, acute kidney injury; eGFR, estimated glomerular filtration rate; ICU, intensive care unit; sCr, serum creatinine.

In Figure 4, we show the association between AKI phenotypes and late kidney dysfunction. In the unadjusted model, CA-AKI was associated with late kidney dysfunction, whereas HA-AKI was not. These associations were confirmed in adjusted models 1 and 2, in which CA-AKI increased the odds of late kidney dysfunction by 9- and 7-fold, respectively (aOR = 9.2, 95% CI: 4.7–17.9 and aOR = 7.3, 95% CI: 3.6–14.8). In the exploratory analysis for AKI severity (use of KRT), no significant association was found in the fully adjusted model 2 (aOR = 2.5, 95% CI: 0.9–6.8). Subgroup analysis by sex revealed a similar pattern of association (Supplementary Table S7).

## Discussion

### Main Findings

In this prospective cohort study, we assessed late kidney dysfunction in survivors of moderate-to-severe COVID-19 hospitalization using a composite outcome. Late kidney dysfunction was present in 1 in 4 survivors of the cohort at the long-term follow-up. After adjusting for confounders, CA-AKI, but not HA-AKI, was an independent risk factor for late kidney dysfunction, indicating that a specific AKI phenotype (CA-AKI) may play a crucial role in the development of late kidney dysfunction. Urinary biomarkers of kidney tubular cell injury were altered in the late kidney dysfunction group at follow-up, suggesting the occurrence of an ongoing kidney structural injury. Overall, our findings showed that renal long COVID was frequent in this cohort of survivors of moderate-to-severe COVID-19 hospitalization and was independently associated with the occurrence of CA-AKI.

### Late Kidney Dysfunction in Long COVID

Previous studies assessing the long-term effects of COVID-19 on the kidney are mostly retrospective, do not include kidney function as the primary outcome, and generally include follow-up periods < 6 months.^34^ Moreover, few studies have simultaneously assessed the long-term changes in eGFR, urinary albumin-to-creatinine ratio, and urine sediment, and/or used adequate thresholds (eGFR < 60 ml/min per 1.73 m^2^) to define late kidney dysfunction.^35^ The present study is unique because it assessed a prospective cohort of COVID-19 survivors, analyzed late kidney dysfunction as the primary outcome, evaluated 2 AKI phenotypes, had a follow-up up to 11 months, investigated several urinary biomarkers, and evaluated a composite outcome.

There are few prospective studies to compare with ours. A study from China prospectively assessed 1734 COVID-19 survivors (median follow-up time 342 days).^36^ The authors found low eGFR (i.e., < 60 ml/min per 1.73 m^2^) in 8.3% at follow-up, which was higher in patients with AKI during hospitalization after multivariate adjustment. The percentage of eGFR reduction varied according to the KDIGO-AKI stages. However, the authors did not assess CA-AKI, urinary albumin-to-creatinine ratio, or kidney urinary biomarkers. Another prospective cohort study compared 443 individuals with mild to moderate COVID-19 (the majority were nonhospitalized) with matched controls for a median of 9.6 months after the first positive COVID-19 test. The authors found that the eGFR slope of patients with COVID-19 was −2.4 ml/min per 1.73m^2^.^37^ In a Swedish COVID-19 cohort of 134,564 individuals, an eGFR slope of −3.4% was observed, being more severe among those who were hospitalized (−5.4%).^18^ The authors compared the eGFR decline in COVID-19 survivors with patients with pneumonia or influenza without COVID-19. The eGFR decline was greater in COVID-19 patients when the full cohort was assessed, but similar in the subgroup analysis of the hospitalized patients. These results of eGFR slope are similar to those found in the present study (−4.8 ml/min per 1.73 m^2^); however, we found that eGFR change differed strikingly between AKI phenotypes, with a greater decline in CA-AKI (−61.9 ml/min per 1.73 m^2^), whereas HA-AKI showed a positive change (42.5 ml/min per 1.73 m^2^).

Our study is the first to identify the impact of different AKI phenotypes with late kidney dysfunction among survivors of COVID-19 hospitalization. Notably, late kidney dysfunction occurred even though almost all patients with CA-AKI recovered their eGFR during hospitalization, and 92% of them were discharged with an eGFR ≥ 60 ml/min per 1.73 m^2^. Patients with CA-AKI were older, mostly male, and presented a higher Charlson comorbidity index and Simplified Acute Physiology Score III score at hospital admission. They used KRT almost twice as frequently as those with HA-AKI. Moreover, the KRT use within 48 hours from admission was significantly higher in the CA-AKI group, indicating a more severe clinical condition at admission. Intriguingly, patients with HA-AKI had worse clinical nonrenal outcomes throughout the hospitalization period, probably associated with a worse pulmonary condition evolution, which was confirmed by the higher intubation rates. Taken together, these findings suggest that patients with CA-AKI were admitted to the hospital in worse clinical conditions and were more vulnerable to an earlier onset of severe AKI, but they experienced better nonrenal outcomes. Moreover, they were discharged with partial recovery of kidney function.

We hypothesize that a lack of or poor health care quality before hospitalization might be a determinant of CA-AKI development and late kidney dysfunction. However, the available data we analyzed showed that there were no significant differences between CA-AKI and HA-AKI groups regarding time from the first COVID-19 symptom to hospitalization, socioeconomic level, and exposure to greenspace. Future research should investigate these exposures to test if poor health care quality significantly impacts AKI phenotypes and later kidney dysfunction. In addition, staging the severity of CA-AKI by using an extended KDIGO definition^30^ may be useful to stratify risk groups for later kidney dysfunction, because in-hospital outcomes vary according to severity.

The independent association between CA-AKI and late kidney dysfunction may reflect a failure to identify patients at high risk for AKI, a delay in an early AKI diagnosis, and a lack of prevention and timely treatment of AKI in the outpatient setting. Consistent with this hypothesis, the adoption of a KDIGO bundle of supportive strategies in patients at high risk for AKI has been shown to significantly reduce its incidence.^38^

A total of 13 patients who did not experience AKI during hospitalization showed late kidney dysfunction at the follow-up visit. These results suggest that the COVID-19 virus may also have a deleterious effect on kidney function in those without AKI during the acute phase of the disease. Angiotensin-converting enzyme receptors are abundantly expressed in the kidneys, potentially facilitating viral entry into kidney tissue. Mice experimentally infected with mouse hepatitis virus-1 coronavirus develop lung and kidney injury similar to that seen after SARS-CoV-2 infection. Treatment with a 15-amino-acid synthetic peptide designed to prevent the binding of spike glycoproteins from SARS-CoV-2 and mouse hepatitis virus-1 with their receptors, specifically angiotensin-converting enzyme 2 and carcinoembryonic antigen-related cell adhesion molecule 1, respectively, prevented early and late kidney expression of profibrotic proteins and biomarkers of tubular injury in the infected mice.^39^

The mechanisms causing the AKI-to-CKD transition remain incompletely understood and have been attributed to a maladaptive cellular repair process after AKI.^40^ The identification of increased levels of clusterin and trefoil factor 3, which are urinary biomarkers associated with kidney tubular epithelial cell damage and CKD progression,^41^^,^^42^ in our cohort of patients with late kidney dysfunction is consistent with this hypothesis.^43^^,^^44^ The characteristics of COVID-19 might trigger specific mechanisms for chronic kidney injury. SARS-CoV-2–related peptides have been shown to induce irreversible endothelial-to-mesenchymal transition in endothelial capillary cells from different organs, including the kidneys.^45^ Persistence of SARS-CoV-2 has been reported in surviving patients up to 4 months after acute infection in diverse tissues, including the kidneys.^46^ Moreover, it has been shown that the formation of damage-associated molecular patterns during COVID-19 triggers persistent low-grade inflammation over time.^47^

### Strengths and Limitations

Our study has strengths. To the best of our knowledge, this is the largest post–COVID-19 cohort being conducted in Latin America, which allowed us to obtain a large sample size in a real-world setting. We evaluated the participants during in-person visits, objectively measuring outcomes of interest, whereas previous studies mainly conducted phone interviews or only examined electronic health records. The available evidence in the literature primarily focused on eGFR and sCr, whereas ours assessed several other kidney function biomarkers.

This study also presents limitations. It was conducted at a single public academic hospital, which served as the primary reference center for COVID-19 in the metropolitan area of São Paulo city, Brazil. Patients included in this study likely represent the most severe cases, and therefore, the findings might lack generalizability to mild-moderate cases. These data represent a prevaccine first COVID wave period, and generalizability to other COVID waves is discouraged. The substantial attrition observed up to the follow-up visit, as expected in this population, may have led to an underestimation of late kidney dysfunction. Individuals who were discharged alive but deceased between discharge and follow-up visits were in a worse clinical status at admission than those who were included (Supplementary Table S3). However, they had less ICU admission and used less orotracheal intubation, possibly representing less severe COVID-19 cases. Importantly, they were older, which is an important factor associated with kidney dysfunction. Although this may introduce survival bias in the estimates, they represent an intrinsic and unavoidable component of studies involving severely diseased patients.

Limitations regarding kidney function that warrant concern include the unavailability of prehospitalization sCr levels, which may have included undiagnosed cases of CKD and/or patients with previous kidney dysfunction. This unavailability may have also impacted the main finding of CA-AKI as a critical risk factor for late kidney dysfunction, because this AKI phenotype occurred before hospital admission, but no data were available regarding its timing. The lack of urinary albumin-to-creatinine ratio and urine sediment assessments at hospital discharge may have led to overdiagnosis at follow-up. Further research from our group will address this gap by evaluating these markers in the already scheduled future prospective follow-up time points. Lastly, there was relevant missing data for urinary biomarkers; however, this missingness was completely at random due to operational procedures.

### Conclusion

Our results demonstrated a high incidence of late kidney dysfunction (i.e., renal long COVID) among survivors of moderate-to-severe COVID-19 hospitalization. This finding suggests that renal long COVID may represent a significant burden for public health in the post-COVID era. Importantly, we identified CA-AKI as an independent risk factor for renal long COVID. Factors associated with CA-AKI are potentially modifiable with the use of adequate protocols to prevent and identify AKI in a timely manner, avoiding or minimizing progression to more severe stages.

Our findings also suggest that kidney function must be closely monitored in survivors of moderate-to-severe COVID-19 hospitalization who experienced CA-AKI. Early assessment of kidney function in these patients will be vital to promptly establish adequate treatment and monitoring strategies, aiming to prevent greater eGFR decline and to delay progression to CKD.^48^

## Appendix

### List of the Members of the HCFMUSP COVID-19 Study Group

Caroline S. Faria, PhD; Laura S. Azevedo; and Fábio Augusto R. Gonçalves.

## Disclosure

EAB received speaker fees from Baxter, AstraZeneca, and Fresenius outside the submitted work. All the other authors declared no competing interests.

## Supplementary Material

Supplementary File (PDF) Assessment of sociodemographic and environmental variables. **Supplementary Methods. Supplementary References. Table S1.** Missing data. **Table S2.** Comparison between survivors who participated and who did not participate in the follow-up in-person visits. **Table S3.** Comparison between survivors who in the follow-up in-person visits and those who died after hospital discharge. **Table S4.** Crude eGFR slope values according to the AKI status. **Table S5.** Characteristics of the cohort according to late kidney dysfunction. **Table S6.** Urinary biomarkers levels in patients with and without late kidney dysfunction. **Table S7.** Subgroup analysis for the association between acute kidney injury phenotypes and late kidney dysfunction by sex. Strengthening the Reporting of Observational Studies in Epidemiology Checklist.

## Footnotes

Supplementary File (PDF)
Assessment of sociodemographic and environmental variables.
**Supplementary Methods.**

**Supplementary References.**
**Table S1.** Missing data. **Table S2.** Comparison between survivors who participated and who did not participate in the follow-up in-person visits. **Table S3.** Comparison between survivors who in the follow-up in-person visits and those who died after hospital discharge. **Table S4.** Crude eGFR change values according to the AKI status. **Table S5.** Characteristics of the cohort according to late kidney dysfunction. **Table S6.** Urinary biomarkers levels in patients with and without late kidney dysfunction. **Table S7.** Subgroup analysis for the association between acute kidney injury phenotypes and late kidney dysfunction by sex. Strengthening the Reporting of Observational Studies in Epidemiology Checklist.
