# Supplementary material for: Community-Acquired Acute Kidney Injury and Late Kidney Dysfunction in Survivors of COVID-19 Hospitalization
Source: Kidney Int Rep. 2025 Jul 3;10(9):3032–43. doi: 10.1016/j.ekir.2025.06.048 (PMC12446965; doi:10.1016/j.ekir.2025.06.048)
Supplement: Supplementary File (PDF) — Assessment of sociodemographic and environmental variables. Supplementary Methods. Supplementary References. Table S1. Missing data. Table S2. Comparison between survivors who participated and who did not participate in the follow-up in-person visits. Table S3. Comparison between survivors who in the follow-up in-person visits and those who died after hospital discharge. Table S4. Crude eGFR slope values according to the AKI status. Table S5. Characteristics of the cohort according to late kidney dysfunction. Table S6. Urinary biomarkers levels in patients with and without late kidney dysfunction. Table S7. Subgroup analysis for the association between acute kidney injury phenotypes and late kidney dysfunction by sex. Strengthening the Reporting of Observational Studies in Epidemiology Checklist. [file mmc1.pdf]

SUPPLEMENTARY MATERIAL

Supplementary Methods

Methods for the analysis of kidney urinary biomarkers

We measured albumin, neutrophil gelatinase-associated lipocalin (NGAL), kidney injury molecule-1 (KIM-1), tissue inhibitor of metalloproteinase 2 (TIMP-2) x insulin-like growth factor–binding protein-7 (IGFBP-7), clusterin, monocyte chemoattractant protein-1 (MCP-1), interleukin-18, glutathione S-transferase, β-2 microglobulin, cystatin C, and intestinal trefoil factor 3. The analysis was performed using LUMINEX-xMAP technology using three kits (Bio-Plex Pro RBM human kidney toxicity PANELS 1 and 2, Bio-Rad Laboratories, Berkeley, California, USA; and MILLIPLEX® MAP Custom Human 2-plex Magnetic Bead Panel, EMD Merck Millipore, Austin, Texas, USA).

Assessment of sociodemographic and environmental variables

A standardized and validated questionnaire for the Brazilian population was applied at follow-up to classify socioeconomic status in accordance with current criteria of the *Associação Brasileira de Empresas de Pesquisa* in six categories (A-most affluent, B1, B2, C1, C2, and DE)[1]. Ethnicity was self-reported according to the Brazilian Institute of Geography and Statistics in white, black, mixed (*Pardo*, a mixed black and white ethnicity), East Asian, or Brazilian indigenous[2]. Environmental exposure to greenspace by using satellite images of the Sao Paulo metropolitan region from 2018 and 2020 was calculated[3].

Supplementary References

S1. Associação Brasileira de Empresas de Pesquisa (ABEP). Socioeconomic class in Brazil, https://www.abep.org/criterio-brasil (2020).

S2. IBGE. 2022 Census: self-reported brown population is the majority in Brazil for the first time. *2022 Census*, https://agenciadenoticias.ibge.gov.br/en/agencia-news/2184-news-agency/news/38726-2022-census-self-reported-brown-population-is-the-majority-in-brazil-for-the-first-time (2023).

S3. Ferreira JC, Moreira TCL, de Araújo AL, et al. Clinical, sociodemographic and environmental factors impact post-COVID-19 syndrome. *J Glob Health* 2022; 12: 05029.

Supplementary Table S1. Missing data.

	Absolute (n)	Relative (%)
Age	0	0.0
Sex	0	0.0
Ethnicity	2	0.3
Socioeconomic status	9	1.4
Body mass index	115	17.6
**Environmental**		
Greenspace	15	2.3
**Comorbidities**		
Hypertension	14	2.1
Diabetes	13	2.0
Obesity	115	17.6
Chronic respiratory disease	14	2.1
Kidney disease	14	2.1
Cancer	45	6.9
Charlson comorbidity index	21	3.2
SAPS 3 score	235	35.9
Duration of symptoms until admission	0	0.0
**Blood biomarkers**		
C-reactive protein	42	6.4
Hemoglobin	6	0.9
Leukocytes	8	1.2
Neutrophils	6	0.9
Lymphocytes	9	1.4
D-dimer	74	11.3
Oxygen saturation	189	28.9
**Kidney function at hospital admission**		
SCr (mg/dl)	0	0.0
eGFR (ml/min/1.73 m^2)	0	0.0
**Outcomes during hospitalization**		
peak SCr (mg/dl)	0	0.0
Hospital length of stay (days)	14	2.1
ICU admission, n (%)	13	2.0
ICU length of stay (days)	13	2.0
Intubation, n (%)	17	2.6
KRT use, n (%)	13	2.0
**Kidney function at hospital discharge**		
SCr (mg/dl)	0	0.0
eGFR (ml/min/1.73 m^2)	0	0.0
eGFR < 60 ml/min/1.73 m^2, n (%)	0	0.0
**Events between discharge and follow-up**		
Vaccine doses	305	46.6
Hospital readmission, n (%)	0	0.0
COVID reinfection, n (%)	0	0.0
**Kidney function at follow-up**		
SCr (mg/dl)	0	0.0
eGFR (ml/min/1.73 m^2)	0	0.0
eGFR change from discharge (ml/min/1.73 m^2)	0	0.0

**Urinary biomarkers at follow-up**		
Albumin (μg/ml)	115	17.6
Creatinine	22	3.4
Protein	19	2.9
Leukocyte	19	2.9
Red blood cells	20	3.1
NGAL	117	17.9
KIM-1	118	18.0
TIMP-2	117	17.9
IGFBP-7	115	17.6
Clusterin	117	17.9
MCP-1	115	17.6
Interleukin-18	152	23.2
Glutathione S-transferase	122	18.6
Calbindin	122	18.6
Osteopontin	115	17.6
β-2 microglobulin	129	19.7
Cystatin C	131	20.0
Intestinal trefoil factor 3	115	17.6

AKI, acute kidney injury; eGFR, estimated glomerular filtration rate; sCr, serum creatinine; ICU, intensive care unit; SAPS 3, Simplified Acute Physiology Score 3; SpO2, peripheral capillary oxygen saturation; IGFBP-7, insulin-like growth factor-binding protein-7; KIM-1, kidney injury molecule 1; MCP-1, monocyte chemoattractant protein-1; NGAL, neutrophil gelatinase-associated lipocalin; TIMP-2, tissue inhibitor metalloproteinase 2; UACR, urinary albumin-to-creatinine ratio; KRT, kidney replacement therapy.

Supplementary Table S2. Comparison between survivors who participated and who did not participate in the follow-up in-person visits.

	**Participants** (n = 655)	**Non-participants** (n = 539)	**p-value**
Age, years	55.3 ± 14.1	54.9 ± 18.5	0.682
Male	397 (53%)	248 (46%)	0.013
Body mass index, kg/m^2	31.6 ± 12.6	29.4 ± 10.5	0.004
Charlson comorbidity index, median [IQR]	3 [2 – 4]	3 [2 – 5]	0.031
Duration of symptoms until admission, median [IQR], days	8 [5 – 11]	7 [5 – 11]	0.039
SAPS 3	58.6 ± 14.1	56.9 ± 14.0	0.123
Duration of hospitalization, median [IQR], days	12 [7 – 23]	10 [6 – 18]	<0.001
ICU stay	445 (59%)	259 (48%)	<0.001
Use of intubation	305 (41%)	148 (28%)	<0.001

IQR, interquartile range; ICU, intensive care unit.; SAPS 3, Simplified Acute Physiology Score 3.

Supplementary Table S3. Comparison between survivors who in the follow-up in-person visits and those who died after hospital discharge.

	**Survivors who participated** (n = 655)	**Dead after discharge** (n = 157)	**p-value**
Age, years	55.3 ± 14.1	66.9 ± 14.9	<0.001
Male	397 (53%)	88 (56%)	0.538
Body mass index, kg/m^2	31.6 ± 12.6	26.1 ± 8.2	<0.001
Charlson comorbidity index, median [IQR]	3 [2 – 4]	5 [4 – 7]	<0.001
Duration of symptoms until admission, median [IQR], days	8 [5 – 11]	5 [3 – 10]	<0.001
SAPS 3	58.6 ± 14.1	66.7 ± 13.9	<0.001
Duration of hospitalization, median [IQR], days	12 [7 – 23]	13 [7 – 20]	0.654
ICU stay	445 (59%)	72 (46%)	0.002
Use of intubation	305 (41%)	38 (24%)	<0.001

IQR, interquartile range; ICU, intensive care unit.; SAPS 3, Simplified Acute Physiology Score 3.

Supplementary Table S4. Crude eGFR slope values according to the AKI status.

Descriptives

Crude eGFR slope

	N	Mean	Std. Deviation	Std. Error	95% Confidence Interval for Mean		Minimum	Maximum
					Lower Bound	Upper Bound		
**no AKI**	141	-3,7069	17,87832	1,50563	-6,6836	-,7302	-65,44	60,87
**community AKI**	230	-28,7485	31,85929	2,10074	-32,8878	-24,6093	-124,07	69,40
**hospital AKI**	284	19,7455	40,41096	2,39795	15,0255	24,4656	-117,27	123,70
**Total**	655	-2,3314	39,84936	1,55704	-5,3888	,7260	-124,07	123,70

AKI, acute kidney injury; eGFR, estimated glomerular filtration rate.

Supplementary Table S5. Characteristics of the cohort according to late kidney dysfunction.

Variables	No kidney dysfunction (n = 471)	Kidney dysfunction (n = 184)	*p*-value
Age (years)	54.7 ± 13.9	58.5 ± 13.1	0.002
Older age (≥ 60 years), n (%)	194 (41)	98 (53)	0.005
Female, n (%)	226 (48)	76 (41)	0.123
**Ethnicity**, n (%)			0.072
White	221 (46)	89 (49)	
Black	57 (12)	33 (18)	
Mixed	183 (40)	56 (31)	
Others*/Not reported	12 (3)	2 (1)	
Low socioeconomic status, n (%)	178 (39)	80 (44)	0.189
Greenspace (%), median [IQR]	32.5 [22.7 – 53.9]	33.5 [23.8 – 53.5]	0.698
**Hospital admission**, median [IQR]			
Duration of symptoms until admission (days)	8 [6 – 11]	7 [5 – 11]	0.123
sCr (mg/dl)	0.9 [0.7 – 1.1]	1.7 [1.2 – 3.2]	<0.001
Charlson comorbidity index	2.7 [1.2 – 4.0]	3.5 [2.0 – 5.0]	<0.001
SAPS 3 score	58 [47 – 68]	59 [48 – 69]	0.595
SpO2 < 90%, n (%)	92 (27)	40 (32)	0.257
Obesity, n (%)	151 (39)	68 (44)	0.360
**Acute kidney injury**, n (%)	128 (27.2)	171 (92.9)	<0.001
Community-acquired	98 (20.8)	132 (71.7)	<0.001
Hospital-acquired	245 (52.0)	39 (21.2)	
**Events during hospitalization**, median [IQR]			
Hospital length of stay (days)	15 [8 – 25]	16 [9 – 25]	0.390
ICU admission, n (%)	313 (67)	126 (69)	0.620
ICU length of stay (days)	10 [6 – 18]	10 [6 – 19]	0.797
Intubation, n (%)	211 (46)	85 (48)	0.669
Corticoid use, n (%)	313 (67)	116 (63)	0.409
peak sCr (mg/dl)	1.4 [1.0 – 3.3]	1.7 [1.2 – 3.2]	0.002
KRT use, n (%)	43 (9)	48 (26)	<0.001
Days on KRT	11 [5 – 20]	13 [5 – 20]	0.914
Days from admission to KRT, n (%)	5 [2 – 8]	2 [1 – 5]	0.003
KRT use within 48 hours from admission, n (%)	11 (26)	27 (56)	0.003
**Kidney function at discharge**			
eGFR < 60, n (%)	139 (30)	8 (4)	<0.001
**Kidney function at follow-up**			
Albuminuria, n (%)	108 (29)	46 (33)	0.359
Abnormal urine sediment, n (%)	92 (20)	38 (31)	0.792

AKI, acute kidney injury; eGFR, estimated glomerular filtration rate; sCr, serum creatinine; KRT, kidney replacement therapy; SpO2, peripheral capillary oxygen saturation; IQR, interquartile range; ICU, intensive care unit.
Low socioeconomic status was classified according to the *Associação Brasileira de Empresas de Pesquisa* (ABEP) scores C2 + DE.
Missing data: albuminuria (n = 115); abnormal urine sediment (n = 19).
[a] includes indigenous and East Asian.

Supplementary Table S6. Urinary biomarkers levels in patients with and without late kidney dysfunction.

	n	Cohort	No kidney dysfunction	Kidney dysfunction	*p*-value
NGAL* (ng/mL)	538	22.0 [4.1 – 55.0]	20.6 [4.4 – 52.9]	24.5 [3.3 – 67.8]	0.383
KIM-1 (ng/mL)	537	0.39 [0.19 – 0.69]	0.39 [0.20 – 0.67]	0.38 [0.18 – 0.76]	0.886
TIMP-2 (ng/mL)	538	17.6 [9.4 – 34.3]	16.8 [9.3 – 33.6]	18.5 [9.4 – 39.5]	0.212
IGFBP-7 (ng/mL)	540	250 [146 – 1046]	250 [142 – 1000]	252 [148 – 1585]	0.712
TIMP-2*IGFBP-7/1000 $(ng/mL)^2$	538	4.4 [1.5 – 37.0]	4.2 [1.5 – 31.8]	4.6 [1.4 – 48.9]	0.398
Clusterin (ng/mL)	538	22.9 [9.0 – 49.7]	22.6 [8.8 – 47.6]	23.4 [9.1 – 63.2]	0.217
MCP-1 (ng/mL)	540	0.24 [0.12 – 0.44]	0.23 [0.11 – 0.42]	0.25 [0.14 – 0.46]	0.127
Interleukin-18 (pg/mL)	503	60.3 [32.0 – 141.1]	67.6 [32.0 – 141.1]	51.0 [28.8 – 130.1]	0.188
Glutathione S-transferase (pg/mL)	533	9.8 [3.6 – 26.1]	9.8 [3.5 – 25.6]	9.8 [4.2 – 28.0]	0.490
β-2 microglobulin (ng/mL)	526	30.7 [6.9 – 101.1]	31.6 [7.0 – 97.3]	28.7 [6.6 – 119.2]	0.633
Cystatin C (ng/mL)	524	14.8 [3.4 – 31.7]	15.0 [3.5 – 33.2]	14.1 [3.3 – 29.4]	0.449
Trefoil factor 3 (ng/mL)	540	617 [115 – 1364]	529 [115 – 1320]	765 [98 – 1759]	0.119

IGFBP-7, insulin-like growth factor-binding protein-7; KIM-1, kidney injury molecule 1; MCP-1, monocyte chemoattractant protein-1; NGAL, neutrophil gelatinase-associated lipocalin; TIMP-2, tissue inhibitor metalloproteinase 2.
Values are reported in median and interquartile range.

Supplementary Table S7. Subgroup analysis for the association between acute kidney injury phenotypes and late kidney dysfunction by sex.

	Adjusted model 1		Adjusted model 2	
	**Odds ratio (95% CI)**	***p*-value**	**Odds ratio (95% CI)**	***p*-value**
**Male** (n = 353)				
No AKI	reference		reference	
Community-acquired AKI	9.34 (3.63 to 24.02)	<0.001	6.54 (2.42 to 17.70)	<0.001
Hospital-acquired AKI	1.24 (0.46 to 3.39)	0.672	2.18 (0.68 to 6.97)	0.188
AKI without KRT	1.20 (0.44 to 3.30)	0.720	2.18 (0.68 to 6.97)	0.188
AKI with KRT	1.64 (0.47 to 5.71)	0.437	2.43 (0.64 to 9.16)	0.191
**Female** (n = 302)				
No AKI	reference		reference	
Community-acquired AKI	10.12 (3.78 to 27.08)	<0.001	8.68 (3.11 to 24.25)	<0.001
Hospital-acquired AKI	1.05 (0.39 to 2.79)	0.925	2.29 (0.72 to 7.31)	0.163
AKI without KRT	1.02 (0.38 to 2.73)	0.973	2.29 (0.72 to 7.31)	0.163
AKI with KRT	1.33 (0.33 to 5.32)	0.684	3.02 (0.63 to 14.56)	0.168

AKI, acute kidney injury; KRT, kidney replacement therapy.
Adjusted model 1 included older age (reference = <60 years), sex (reference = female), ethnicity (reference = white), socioeconomic status (reference = high + medium), Charlson comorbidity index (continuous), greenspace (continuous), and sCr at hospital admission (continuous).
Adjusted model 2 included model 1 *in addition to* ICU admission (reference = no admission), hospital length of stay (continuous), use of KRT (reference = no use), and eGFR at discharge (continuous).
In the KRT analysis, we stratified the cohort into no AKI (reference), AKI without use of KRT, and AKI with use of KRTI. For this analysis, CA-AKI was included as a covariate in model 1 (reference = no AKI + hospital-acquired AKI), while KRT as covariate was excluded from model 2.

STROBE Statement—Checklist of items that should be included in reports of ***cohort studies***

	Item No	Recommendation	Page No
**Title and abstract**	1	(*a*) Indicate the study's design with a commonly used term in the title or the abstract	1
		(*b*) Provide in the abstract an informative and balanced summary of what was done and what was found	2
**Introduction**			
Background/rationale	2	Explain the scientific background and rationale for the investigation being reported	4
Objectives	3	State specific objectives, including any prespecified hypotheses	4
**Methods**			
Study design	4	Present key elements of study design early in the paper	4
Setting	5	Describe the setting, locations, and relevant dates, including periods of recruitment, exposure, follow-up, and data collection	5
Participants	6	(*a*) Give the eligibility criteria, and the sources and methods of selection of participants. Describe methods of follow-up	5
		(*b*) For matched studies, give matching criteria and number of exposed and unexposed	NA
Variables	7	Clearly define all outcomes, exposures, predictors, potential confounders, and effect modifiers. Give diagnostic criteria, if applicable	5-6
Data sources/ measurement	8*	For each variable of interest, give sources of data and details of methods of assessment (measurement). Describe comparability of assessment methods if there is more than one group	5-7
Bias	9	Describe any efforts to address potential sources of bias	7-9
Study size	10	Explain how the study size was arrived at	7
Quantitative variables	11	Explain how quantitative variables were handled in the analyses. If applicable, describe which groupings were chosen and why	7-8
Statistical methods	12	(*a*) Describe all statistical methods, including those used to control for confounding	7-9
		(*b*) Describe any methods used to examine subgroups and interactions	8-9
		(*c*) Explain how missing data were addressed	7
		(*d*) If applicable, explain how loss to follow-up was addressed	NA
		(*e*) Describe any sensitivity analyses	8-9
**Results**			
Participants	13*	(a) Report numbers of individuals at each stage of study—eg numbers potentially eligible, examined for eligibility, confirmed eligible, included in the study, completing follow-up, and analysed	9
		(b) Give reasons for non-participation at each stage	9
		(c) Consider use of a flow diagram	9
Descriptive data	14*	(a) Give characteristics of study participants (eg demographic, clinical, social) and information on exposures and potential confounders	9-10
		(b) Indicate number of participants with missing data for each variable of interest	9-10
		(c) Summarise follow-up time (eg, average and total amount)	9-10
Outcome data	15*	Report numbers of outcome events or summary measures over time	11-12

Main results	16	(*a*) Give unadjusted estimates and, if applicable, confounder-adjusted estimates and their precision (eg, 95% confidence interval). Make clear which confounders were adjusted for and why they were included	11-12
		(*b*) Report category boundaries when continuous variables were categorized	11-12
		(*c*) If relevant, consider translating estimates of relative risk into absolute risk for a meaningful time period	NA
Other analyses	17	Report other analyses done—eg analyses of subgroups and interactions, and sensitivity analyses	11-12
**Discussion**			
Key results	18	Summarise key results with reference to study objectives	12-13
Limitations	19	Discuss limitations of the study, taking into account sources of potential bias or imprecision. Discuss both direction and magnitude of any potential bias	15-16
Interpretation	20	Give a cautious overall interpretation of results considering objectives, limitations, multiplicity of analyses, results from similar studies, and other relevant evidence	16-17
Generalisability	21	Discuss the generalisability (external validity) of the study results	16-17
**Other information**			
Funding	22	Give the source of funding and the role of the funders for the present study and, if applicable, for the original study on which the present article is based	17

*Give information separately for exposed and unexposed groups.

Note: An Explanation and Elaboration article discusses each checklist item and gives methodological background and published examples of transparent reporting. The STROBE checklist is best used in conjunction with this article (freely available on the Web sites of PLoS Medicine at http://www.plosmedicine.org/, Annals of Internal Medicine at http://www.annals.org/, and Epidemiology at http://www.epidem.com/). Information on the STROBE Initiative is available at http://www.strobe-statement.org.
